# Using Motor Tempi to Understand Rhythm and Grammatical Skills in Developmental Language Disorder and Typical Language Development

**DOI:** 10.1162/nol_a_00082

**Published:** 2023-01-18

**Authors:** Enikő Ladányi, Michaela Novakovic, Olivia A. Boorom, Allison S. Aaron, Alyssa C. Scartozzi, Daniel E. Gustavson, Rachana Nitin, Peter O. Bamikole, Chloe Vaughan, Elisa Kim Fromboluti, C. Melanie Schuele, Stephen M. Camarata, J. Devin McAuley, Reyna L. Gordon

**Affiliations:** Department of Otolaryngology—Head & Neck Surgery, Vanderbilt University Medical Center, Nashville, TN; Department of Linguistics, University of Potsdam, Potsdam, Germany; Department of Pharmacology, Northwestern University Feinberg School of Medicine, Chicago, IL; Department of Hearing and Speech Sciences, Vanderbilt University Medical Center, Nashville, TN; Department of Speech-Language-Hearing: Sciences and Disorders, University of Kansas, Lawrence, KS; Department of Speech, Language and Hearing Sciences, Boston University, Boston, MA; Vanderbilt Genetics Institute, Vanderbilt University, Nashville, TN; Institute for Behavioral Genetics, University of Colorado Boulder, Boulder, CO; Vanderbilt Brain Institute, Vanderbilt University, Nashville, TN; Department of Anesthesiology and Perioperative Medicine, Oregon Health & Science University, Portland, OR; Department of Psychology, Michigan State University, East Lansing, MI; Department of Hearing and Speech Sciences, Vanderbilt University School of Medicine, Nashville, TN; Vanderbilt Kennedy Center, Vanderbilt University Medical Center, Nashville, TN

**Keywords:** developmental language disorder, entrainment, grammar, neural oscillations, rhythm, tapping

## Abstract

Children with developmental language disorder (DLD) show relative weaknesses on rhythm tasks beyond their characteristic linguistic impairments. The current study compares preferred tempo and the width of an entrainment region for 5- to 7-year-old typically developing (TD) children and children with DLD and considers the associations with rhythm aptitude and expressive grammar skills in the two populations. Preferred tempo was measured with a spontaneous motor tempo task (tapping tempo at a comfortable speed), and the width (range) of an entrainment region was measured by the difference between the upper (slow) and lower (fast) limits of tapping a rhythm normalized by an individual’s spontaneous motor tempo. Data from *N* = 16 children with DLD and *N* = 114 TD children showed that whereas entrainment-region width did not differ across the two groups, slowest motor tempo, the determinant of the upper (slow) limit of the entrainment region, was at a faster tempo in children with DLD vs. TD. In other words, the DLD group could not pace their slow tapping as slowly as the TD group. Entrainment-region width was positively associated with rhythm aptitude and receptive grammar even after taking into account potential confounding factors, whereas expressive grammar did not show an association with any of the tapping measures. Preferred tempo was not associated with any study variables after including covariates in the analyses. These results motivate future neuroscientific studies of low-frequency neural oscillatory mechanisms as the potential neural correlates of entrainment-region width and their associations with musical rhythm and spoken language processing in children with typical and atypical language development.

## INTRODUCTION

### Developmental Language Disorder

The most characteristic errors in children with developmental language disorder (DLD) are in expressive morphosyntax, but lexical and phonological problems can appear as well, and comprehension can be impaired in addition to production (Bishop et al., 2016; Leonard, 2014). The linguistic problems in children with DLD cannot be attributed to obvious global impairments in other cognitive domains or peripheral deficits, an intellectual disability, neurological disorders, trauma, emotional or social deficits, or environmental deprivation (Bishop et al., 2016; Leonard, 2014). Impairments have also been found in nonlinguistic domains such as in auditory processing (Leonard, 1998; Tallal & Piercy, 1973), working memory (Gathercole & Baddeley, 1990), motor impairments (Hill, 2001), procedural learning (Ullman & Pierpont, 2005), and statistical learning (Hsu & Bishop, 2011).

Interestingly, recent studies have reported impairment in selected rhythm abilities in children with DLD compared to typically developing (TD) children (Corriveau & Goswami, 2009; Cumming et al., 2015). Beyond relative weaknesses in rhythm discrimination tasks, children with DLD also show difficulties with tapping accurately in synchrony with the beat of musical excerpts/a metronome (Corriveau & Goswami, 2009; Cumming et al., 2015). When asked to continue tapping an isochronous rhythm after a stimulus stops (continuation tapping task), children with DLD performed as consistently as TD children (except at a 667 ms rate; Corriveau & Goswami, 2009; Vuolo et al., 2017; Zelaznik & Goffman, 2010). However, children with DLD tapped at a faster tempo compared to TD children at preschool age (Vuolo et al., 2017). This difference did not appear in slightly older children (Zelaznik & Goffman, 2010). It is, thus, unclear if there is a difference in the tapping tempo in children with DLD versus TD children when there is no external stimulus, such as in continuation tapping or spontaneous (preferred) tapping tasks.

Although we are not aware of any studies measuring spontaneous tapping without external pacing stimulus in children with DLD, a recent paper has shown that faster spontaneous (preferred) tapping rate was one of the main predictors of developmental dyslexia (Bégel et al., 2022), a disorder often comorbid with DLD (Catts et al., 2005). Moreover, individuals with ADHD (another disorder often comorbid with DLD; Tirosh & Cohen, 1998), have shown more variability when they were asked to tap at a comfortable rate without an external pacing stimulus (i.e., at their spontaneous motor tempo, or SMT) compared to typically developing individuals (Kliger Amrani & Zion Golumbic, 2020).

In line with results on impaired rhythm abilities in DLD and converging evidence from various other speech and language disorders as well as from typically developing populations, the Atypical Rhythm Risk Hypothesis (Ladányi et al., 2020) posits that individuals with atypical rhythm are at higher risk for developmental speech/language disorders. One prediction of the Atypical Rhythm Risk Hypothesis is that children with DLD will display various atypical rhythm traits (e.g., impaired rhythm perception, difficulty synchronizing with a beat) due to shared genetic architecture and neural mechanisms underlying musical rhythm and grammar abilities.

The neurobiological underpinnings of potentially shared rhythm and language impairments in DLD are not yet well understood due to the lack of neurobiological studies on this specific question and the very limited neurobiological research on DLD in general. Previous studies have found structural and functional abnormalities in language-related areas such as the inferior frontal gyrus, the superior temporal gyrus, and the striatum (Krishnan et al., 2016). Plante et al. (2017) found similar language network architecture for adults with DLD and TD during an implicit language learning task, though the network showed an increased degree of activation in DLD versus TD. There are thus far only inconsistent findings on abnormal cerebral lateralization in the language-related areas with some supporting (Badcock et al., 2012; de Guibert et al., 2011) and some contradicting evidence (Wilson & Bishop, 2018; see Mayes et al., 2015). Importantly from the perspective of the current article, there is preliminary evidence for structural and functional abnormalities in the striatum (Krishnan et al., 2016), which has a crucial role in language learning as well as in rhythm perception, especially in predicting the beat (Grahn & Rowe, 2013). While several previous studies suggested altered neurobiological processes underlying language processing in DLD, recent work has shown that children with DLD activate the same brain areas to a similar extent as TD children when task difficulty is also appropriate for children with DLD (Krishnan et al., 2021).

### Rhythm and Language Processing

Beyond potential rhythm deficits in children with DLD, there is accumulating evidence that rhythm and language processing are tightly linked in individuals with typical language development (Carr et al., 2014; Gordon et al., 2015; Lee et al., 2020; Patel, 2003; Persici et al., 2021; see Fiveash et al., 2021, and Nayak et al., 2022, for reviews). The mechanisms underlying these associations, however, are not yet well understood. One influential perspective that provides some theoretical traction is dynamic attending theory (DAT; Jones & Boltz, 1989; Large & Jones, 1999). DAT proposes that rhythms in the environment (i.e., stimulus rhythms) serve to entrain (synchronize) intrinsic oscillations in attentional rhythms such that peaks in attention become aligned with rhythmically expected time points in the stimulus (e.g., the speech signal in the domain of language). DAT makes the general prediction that stimulus events that occur at expected time points are better processed than stimulus events that occur at unexpected time points (e.g., early or late). DAT, thus, emphasizes entrained oscillations at multiple hierarchical levels as the link between the processing of rhythm and language. Moreover, one further possibility is that individual differences in rhythm and spoken language processing may be at least partially accounted for by individual differences in the characteristics of the underlying intrinsic oscillations. Conceptually, two such characteristics, emphasized in extensions of DAT (McAuley et al., 2006), are an individual’s preferred tempo and their entrainment-region width—corresponding to the relative range of tempi that afford attentional entrainment.

### Preferred Tempo and Entrainment Region

**Figure 1.**
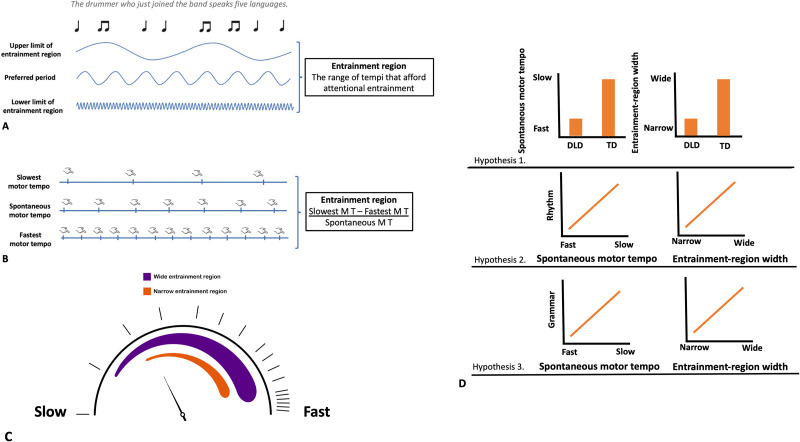
Visual summaries of concepts and predictions of the current study. (**A**) Oscillators at different hierarchical levels that support the processing of musical rhythm and spoken linguistic stimuli. Entrainment region is the range of tempi that afford attentional entrainment. Individuals are able to process regularities only at tempi that fall within their entrainment region. (**B**) We measured the upper limit of the entrainment region with slowest motor tempo, preferred tempo with spontaneous motor tempo, and the lower limit of the entrainment region with fastest motor tempo. Entrainment-region width was calculated as the difference between individuals’ slowest and fastest motor tempi normalized by their spontaneous motor tempo. (**C**) Individuals with wide entrainment region (purple) have a high upper limit and low lower limit of entrainment region and are able to process regularities at very slow and very fast tempi. Individuals with a narrow entrainment region (orange) have a reduced upper and/or lower limit of entrainment region and cannot process regularities at very slow and/or very fast tempi. (**D**) Visual summary of the predictions of the current study.

The perceived organization of sound patterns is characterized by multiple factors, including the perceived beat and meter. Beat and metrical processing can appear only within a limited time range, that is, if the beats appear with approximately 100 ms to 2,500–3,000 ms intervals between beats (Fraisse, 1963; McAuley, 2010; Pöppel, 1997), with precise and predictive temporal synchronization (tapping to the beat) between 250 ms and 1,000 ms (Repp & Su, 2013). In the context of DAT, individual differences in this tempo range are proposed to be influenced by two factors: the preferred period (P_0_), which is the period of a latent intrinsic oscillator that reflects an individual’s preferred tempo and the entrainment region (McAuley et al., 2006; Figure 1A and 1C). Preferred tempo and entrainment-region width would, thus, fundamentally influence individuals’ rhythm abilities since beats and metrical structures appearing at a time span exceeding one’s entrainment-region width cannot be perceived by the individual. In the context of speech–language processing, preferred tempo and entrainment-region width would influence the time range in which dynamic attending can efficiently support predictive processes that were proposed to play a role at multiple levels of spoken language processing.

One method that has been used to estimate an individual’s preferred tempo is assessing an individual’s preferred tapping tempo, referred to as their SMT (Drake et al., 2000; McAuley et al., 2006; Michaelis et al., 2014; cf. Kliger Amrani & Zion Golumbic, 2020; Figure 1C). Unpaced tapping tasks also have been used to estimate the width (tempo range) of the entrainment region by asking participants to tap a regular rhythm as slowly as they can (slowest motor tempo) or as fast as they can (fastest motor tempo) and then either taking the absolute difference between slowest and fastest motor tempo (Drake et al., 2000) or the difference normalized by dividing by SMT (McAuley et al., 2006; Figure 1B).

Similarly, Drake et al. (2000) emphasized that fastest and slowest motor tempi can provide an indication of the upper and lower limits of the range in which we are able to process regularities and integrate events into a sequence. They suggest that lower frequency (slow) oscillators make children able to process higher hierarchical levels in music (metrical structure) or spoken language (syntactic structure) by allowing for temporal prediction and the generation of expectancies based on higher-order structures. Higher frequency (fast) oscillators, on the other hand, are focused on levels lower than the preferred tempo and are involved in processing regularities appearing at a fast tempo (e.g., processing the features of tones in music and phonemes and syllables in speech), enabling local processing.

Although the neural correlates of preferred tempo and entrainment-region width are not yet well understood, recent work has shown that hierarchically organized neural oscillations across the gamma, beta and delta frequency ranges could be the neural basis of oscillatory dynamics proposed by entrainment theories (Tal et al., 2017; see Large et al., 2015, for a summary). Beyond auditory areas (superior temporal gyrus), motor regions (supplementary motor areas) including subcortical structures (cerebellum, striatum) have been proposed to be involved in beat processing (Cannon & Patel, 2021; Grahn & Brett, 2007; Kasdan et al., 2022; Merchant et al., 2015). Results from the current study will help to outline testable hypotheses for future neurobiological studies on the role of endogenous oscillations in musical rhythm and spoken language processing in typical and atypical language development.

### The Development of Preferred Tempo and Entrainment Region

McAuley et al. (2006) proposed two hypotheses about developmental changes in individuals’ preferred tempo and entrainment-region width. The preferred period hypothesis proposes that the preferred tempo of event tracking is relatively fast in childhood and continuously becomes slower throughout childhood, adolescence, and adulthood. The entrainment region hypothesis proposes that the range of tempi that afford stable entrainment is narrow at the beginning of life, then gets broader until adulthood, and becomes narrower again at late adulthood. These changes in the width of entrainment region result from (1) the lower (fast) limit of entrainment region getting gradually faster (enabling processing of faster tempi) with age throughout childhood and then slowing again in late adulthood, and (2) the upper (slow) limit of the entrainment region becoming slower throughout childhood (enabling the processing of slower tempi) and then increasing in tempo again in late adulthood.

Results from a large-scale study by McAuley et al. (2006) in individuals ages 4 through 95 as well as converging evidence from several other studies using various methods, also support both hypotheses (Drake et al., 2000; Provasi & Bobin-Bègue, 2003; Vanneste et al., 2001). Drake et al. (2000) found that in a sample of adults and 4-, 6-, 8-, and 10-year-old children, SMT became increasingly slower and entrainment region got wider with age, while Provasi and Bobin-Bègue (2003) found faster SMT in 2.5- and 4-year-olds than previous studies had shown in adults.

These developmental changes in preferred tempo and entrainment-region width have been proposed to contribute to the development of both metrical processing in music (McAuley et al., 2006) and spoken language (Drake et al., 2000). To our knowledge, however, associations between preferred tempo and entrainment-region width with language skills have not been investigated, nor have potential differences in preferred tempo and the width of entrainment region between children with typical and atypical language development.

### Current Study

The current study extends previous work by considering potential differences between children with DLD and TD children in their preferred tempo and width of an entrainment region, and the association of these characteristics with rhythm and language skills. Thus, an overarching aim of the study was to integrate approaches from the literature on rhythm skills in children with DLD with the preferred period and entrainment-region hypotheses. We propose that the development of preferred tempo and entrainment-region width is delayed but follows a similar pattern in children with DLD compared to their same-aged TD peers and that this developmental delay is associated with lower rhythm aptitude and expressive grammar skills. We therefore evaluated group differences between TD children and children with DLD in preferred tempo and entrainment-region width as well as associations of preferred tempo and entrainment-region width with rhythm aptitude and expressive grammatical ability.

As discussed above, the upper (slow) and lower (fast) limit of an entrainment region measured by slowest and fastest motor tempo could be also relevant in characterizing individuals’ temporal processing (Drake et al., 2000). Therefore, beyond investigating entrainment-region width with a normalized measure, we will also examine separately the upper (slow) and lower (fast) limits of entrainment region with absolute measures in follow-up analyses. The upper limit of the entrainment region, measured by slowest motor tempo, has been proposed as a correlate of processing of information at high hierarchical levels (syntactic/metrical structure); therefore, it could be especially relevant in the context of children with DLD, who show characteristic impairments in syntactic processing. The lower limit of the entrainment region, measured by fastest motor tempo, has been posited as a correlate of processing of information at low hierarchical levels (sounds/tones); therefore, it could be also reduced in children with DLD who are often also characterized by phonological deficits (Ramus et al., 2013). That is, the subset of children with DLD who are not able to tap as fast may have poorer fine-grained temporal processing of speech associated with their phonological deficits. There is prior evidence for impairments in several aspects of phonological processing and production in DLD including discrimination between highly similar phonemes (Leonard et al., 1992), phoneme and syllable production (Bortolini & Leonard, 1996; Maillart & Parisse, 2006), and acquiring novel word forms due to impaired encoding (McGregor et al., 2020). Suboptimal perception of phonemes and syllables could be directly related to restricted lower limit of the entrainment region while other phonological problems may have a more indirect or no relationship with the lower limit of the entrainment region.

There were three main hypotheses concerning differences in preferred tempo and the entrainment-region width between TD children and those with DLD, and the more general relation of preferred tempo and entrainment-region width with rhythm aptitude and grammar.

The first hypothesis is that *children with DLD are delayed in the development of rhythm abilities compared to TD children*. As preferred tempo has been shown to become slower and entrainment region wider in childhood with increased age (Drake et al., 2000; McAuley et al., 2006), we predicted that children with DLD will show a faster preferred tempo and narrower entrainment region than TD children of a similar age. We measured SMT as an index of preferred tempo and the difference between the fastest and slowest possible tapping tempo divided by SMT as an index for entrainment-region width following McAuley et al. (2006).

The second hypothesis is that both *preferred tempo and width of the entrainment region are related to rhythm aptitude*. We predicted that children across the TD and DLD groups with slower preferred tempo and wider entrainment region will show more accurate performance on a rhythm aptitude test.

The third hypothesis is that both *preferred tempo and the width of the entrainment region are related to grammar skills*. We predicted that children across the TD and DLD groups with slower preferred tempo and wider entrainment region will show more accurate performance on the expressive grammar task.

## MATERIALS AND METHODS

### Participants

Data from 16 children with DLD and 114 TD children were analyzed in the current article. (See characteristics of the two groups in Table 1.) Note that all children were between ages 5 and 7 when they were screened and enrolled in the study and underwent initial language testing, but one child in each group turned 8 by the time they completed their participation in the tapping experiment (the study was completed over multiple visits to the lab). An additional 20 children (18 TD, 2 DLD) completed the tapping tasks, but their results were excluded prior to data analysis due to inadequate task performance (faster/slower SMT than fastest/slowest motor tempo or did not follow instructions (*n* = 18), technical error (*n* = 1) or experimenter error (*n* = 1)).

**Table 1.** Means (standard deviations) and comparison of group characteristics in the TD and DLD groups.

Measure	TD	DLD	Difference
*N*	114	16	
Female	66	4	
Age	6.57 (0.85)	6.68 (0.87)	*F*(1, 128) = 0.24, *p* = 0.625
Socioeconomic status^a^	7.48 (0.99)	7.00 (1.04)	*F*(1, 123) = 2.87, *p* = 0.093
PTONI standard score^b^	121.73 (18.94)	100.81 (10.88)	*F*(1, 120) = 18.50, *p* < 0.001
TOLD-P:4 Picture Vocabulary^c^	12.86 (2.36)	10.00 (2.92)	*F*(1,1 28) = 19.38, *p* < 0.001
TOLD-P:4 Sentence Imitation^c^	12.59 (2.05)	7.88 (2.09)	*F*(1, 127) = 73.78, *p* < 0.001
TOLD-P:4 Morphological Completion^c^	12.80 (1.74)	7.94 (2.84)	*F*(1, 128) = 91.98, *p* < 0.001
TOLD-P:4 Spoken Language Index^d^	114 (10)^e^	88 (11)	*F*(1, 76) = 75.27, *p* < 0.001

*Note*. TD = typically developing, DLD = developmental language disorder, PTONI = Primary Test of Nonverbal Intelligence, TOLD = Test of Language Development.

^a^ Range: 1–9: 1 corresponds to no education, 9 corresponds to 3–4+ years of graduate or professional school. The group average, 7, corresponds to 3–4 years of college/technical school.

^b^ Scaled scores, range: 70–130. Scores ≥78 indicate no evidence of global intellectual disability.

^c^ Scaled scores, range: 1–20; 8–12 corresponds to average performance, with scores <8 reflecting below average performance.

^d^ Scaled scores, range: 42–159. Scores <95 on this or other indices were used to confirm the presence of DLD in addition to clinical judgment. Individual performance of children with DLD on each TOLD index is provided in Table S1 in the Supporting Information.

^e^ Note that average performance on the Spoken Language Index in the TD group was calculated based on the performance of 62 participants who completed all TOLD subtests.

Briefly, the eligibility criteria were as follows: We enrolled children with English as their primary language and spoken at home, without genetic or neurological disorders or hearing problems, with minimal or no symptoms of autism spectrum disorder as measured by the Childhood Autism Rating Scale, Second Edition—Standard Version (CARS; Schopler et al., 2010), with a nonverbal IQ greater than or equal to a standard score of 78, and without significant articulation deficits. Children were assigned to the DLD group based on a clinical best estimate of a speech-language pathologist or supervised trainee and performance on standardized language tests (see details below).

Among the 130 children with usable tapping scores, some children did not have valid rhythm discrimination (*n* = 2 DLD and *n* = 6 TD) or expressive grammar (*n* = 2 DLD and *n* = 2 TD) data. Missing data were excluded in a pairwise manner from the analyses.

Ethical approval was obtained from the Vanderbilt University Institutional Review Board, and parents gave written informed consent for participation and children gave verbal assent at each visit. We compensated the time and effort of families with gift cards, and children received a small toy at each visit. Study data were managed and stored using REDCap electronic data capture tools (Harris et al., 2009).

### Recruitment and Screening

Participants of the study were part of the Rhythm and Grammar cohort (Nitin et al., in press), a group of children tested in Nashville between 2016 and 2019 on a large battery of language, music, and cognitive tasks. Participants who completed the tapping task were selected as potential participants for the current study.

Children with English as their primary language spoken at home and without genetic or neurological disorders were recruited for the Rhythm and Grammar cohort from multiple sources in the Middle Tennessee community, including schools, libraries, after-school care centers, museums, and mailing lists. Fifty-six children were screened as potential DLD participants and 147 children were screened as potential TD participants (see detailed description of reasons for excluding screened children below).

A screening visit (approximately 2 hr) was conducted in a quiet room on the campus to determine study eligibility and TD or DLD group assignment. Participants completed a hearing screening (bilateral pure-tone audiometry at 20 dB SPL at 1000, 2000, and 4000 Hz in each ear); a nonverbal intelligence test (Primary Test of Nonverbal Intelligence (PTONI); Ehrler & McGhee, 2008); a screener for autism (CARS; Schopler et al., 2010); a language assessment (a set of subtests of the Test of Language Development—Primary: 4th edition (TOLD-P:4); Newcomer & Hammill, 2008); and the phonological probe and screening probe from the Test of Early Grammatical Impairment (TEGI; Rice & Wexler, 2001). Children who were screened for the DLD group completed all subtests from the TOLD-P:4, whereas in the case of the TD group, typical language development was confirmed with the Picture Vocabulary, Sentence Imitation, and Morphological Completion subtests of the TOLD-P:4.

For the purposes of this article, low language performance on one or more clinical assessments was used as a proxy for DLD. All participants classified as DLD were characterized by speech-language pathologists at our lab as displaying expressive and/or receptive language disorders based on clinical judgment. In addition, DLD participants must have scored below 95 on one or more subtests on the TOLD-P:4. (Note that although eligibility for special education services for speech and language disorders often requires quotients one or more deviations below norm referenced means, there have been a number of recent articles arguing that these cutoffs may in fact underidentify children with clinically and/or educationally significant language disorders (see, e.g., Spaulding et al., 2006).) The scores for each participant with DLD are provided in Table S1 in the Supporting Information. Criterion-referenced Screening Probe results from the TEGI, indicating whether children scored below or at/above criterion, are also provided in the table, as the TEGI has been shown to reliably identify children at risk for DLD (Weiler et al., 2018). Average performance on the Spoken Language Index in the DLD group and a subset of the TD group, that completed all TOLD subtests, is provided in Table 1. Of the 56 children screened as potential DLD participants, 34 participants did not meet DLD eligibility due to one or more of the following reasons: concern for autism spectrum symptoms, behavioral challenges, <78 standard score on PTONI, significant articulation deficits such that morphosyntactic forms such as plural or past tense markers could not be accurately assessed, typical language performance during the screening visit, or a failed hearing screening. We, therefore, had 22 eligible children with DLD after screening.

Children were assigned to the TD group if they scored average or above on the Picture Vocabulary, Sentence Imitation, and Morphological Completion subtests of the TOLD-P:4 and did not display characteristics of DLD as judged by SLP clinicians. Note that one TD participant did not complete the TOLD-P:4 Sentence Imitation subtest due to non-compliance. We decided to include the child in the final sample based on their performance on other tasks and expert clinical opinion.

Based on the above exclusion and inclusion criteria, 22 children were assigned to the DLD group and 137 children were assigned to the TD group. These participants were invited back to complete multiple tasks measuring rhythm and language skills over 1–2 visits. Only the relations between tapping and select measures are the focus of the current article; results on other tasks are reported elsewhere (Nitin et al., in press). Altogether *n* = 18 DLD (5 female, *M*_Age_ = 6.77, *SD*_Age_ = 0.88) and *n* = 132 TD (75 female, *M*_Age_ = 6.51, *SD*_Age_ = 0.84) of the eligible children participated in the study visits, and completed the tapping tasks. After excluding children with inadequate task performance or technical/experimental errors (see above), the final sample comprised *N* = 16 children with DLD and *N* = 114 children with TD.

### Procedures

#### Tapping task

**Figure 2.**
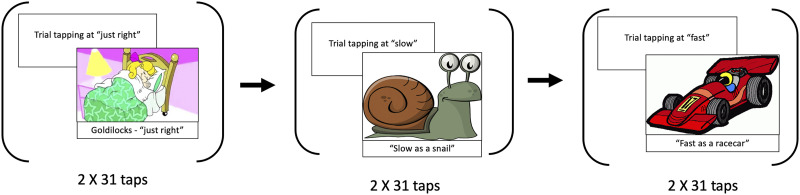
Summary of the tapping task. First, children were asked to tap at a comfortable tempo, then at a slow tempo, and finally at a fast tempo.

The rhythmic tapping task had three conditions (SMT, fastest motor tempo, slowest motor tempo), following the design of McAuley et al. (2006; see also Drake et al., 2000, for a similar design). The child was seated in front of an iPad, which they tapped with one finger throughout the assessment. Children were first given two opportunities to practice tapping on the screen while the test administrator ensured that the taps were being registered by the iPad. After the practice round, the administrator played a slideshow on a separate screen accompanied with verbal instructions on how to “tap” (Figure 2). The first slide was on the story about Goldilocks who “prefers a bed that is not too soft and not too hard, but feels just right.” Children were instructed to tap at a pace that is “just right” for them. After the “just right” tempo participants were shown a snail on the presentation screen and were instructed to tap as “slowly as they can just like a snail.” Finally, they were shown a racecar and instructed to “tap as fast as they can, just like a racecar.” The children performed two rounds of tapping at each of the just right, slow, and fast tempi, with 31 taps in each round. The experimenter instructed the child to stop tapping when the child completed the 31 taps. The number of taps was counted by the software and indicated with a progress bar on the iPad screen. Each of the conditions was preceded by a practice phase, to ensure the participants were following instructions correctly. Custom software was used to record the taps.

Inter-tap intervals were retrieved from the program, and data were processed with R software (Version 3.6.0; R Core Team, 2017). Inter-tap intervals below 50 ms were removed from the data as these could result only from a technical error or a finger bounce. Medians and standard deviations of each run were calculated, and the values for the two runs were averaged in each condition. SMT was used as a proxy for preferred tempo, the difference between slowest and fastest motor tempo normalized by SMT were used as a proxy for entrainment-region width. Slowest and fastest motor tempi were used as measures of upper and lower limits of entrainment region.

#### The validity of SMT as a measure of preferred tempo: Correlations with preferred perceptual tempo in a representative subset of participants

The idea that SMT is a valid estimate of preferred tempo is supported by previous studies showing strong associations between SMT and preferred perceptual tempo (measured by tempo judgments; McAuley, 2010; McAuley et al., 2006). To confirm that this association was present for the participant sample in this study, a subset of children representative of the whole sample’s age, DLD status, and sex characteristics (*n* = 20, 12 female, 3 DLD, aged *M* = 6.87 yr, *SD* = 0.86) completed a preferred perceptual tempo task in addition to the tapping task. One additional participant completed the preferred perceptual tempo task, but their data were excluded before data analysis as they were not able to follow instructions.

In the preferred perceptual tempo task, children were presented with isochronous monotone sequences at different tempi, and they were asked to rate the tempo of the sequences. A physical board with values ranging from −10 to 10 was used to mark the tempo of each sequence. Negative values meant slow tempo with −10 being “too slow,” positive values meant fast tempo with 10 being “too fast,” and 0 meant “just right.” Slow tempo was represented on the board with a turtle figure, while fast tempo was represented with a rabbit figure. The seed tempo was determined based on each child’s SMT, which was assessed prior to the Preferred Perceptual Tempo task. Children completed 22 trials; two trials matched their SMT, 10 trials were slower, and 10 trials were faster than their SMT (see a detailed description of the task in McAuley et al., 2006). Two pseudo-random orders of stimuli were created, and their presentation was counterbalanced across children. There was a strong positive correlation between SMT and preferred perceptual tempo (*r* = 0.80, *p* < 0.001), supporting the use of SMT as a measure of preferred tempo.

#### Primary Measures of Music Audiation (PMMA), Rhythm section

Rhythm aptitude was measured with the rhythm section of the computer-based version of the Primary Measures of Music Audiation (Gordon, 1979). In each trial, the children were presented with two monotonic melodic rhythmic excerpts that were either identical or slightly varied in their temporal pattern, and then they were asked to decide whether the two rhythms were the same or different. The task consisted of two practice trials with one same and one different trial and 40 test trials with 20 same and 20 different trials. Children were given feedback about the correctness of their answers only in the practice phase and not in the testing phase. Same and different trials appeared in the same order for each child. The task was implemented in a game format in which a dog named Molly gets one step closer to her home with every answer. Depending on their computer abilities, children responded verbally or by selecting the option with a computer mouse. We used *d*′, the difference between *z*-scored hit and false alarm rate as a measure of rhythm discrimination.

#### Structured Photographic Expressive Language Test (SPELT-3)

Expressive grammatical abilities were measured with the Structured Photographic Expressive Language Test (SPELT-3; Dawson et al., 2003). Children were shown 53 photographs, and the experimenter asked a question about each photograph. The questions are phrased with the aim of eliciting answers containing certain morphosyntactic constructions (e.g., irregular past tense verbs, relative clauses, or reflexive pronouns). Testing the production of multiple syntactic structures allows for a reliable assessment of individual differences in children’s expressive grammatical abilities with a wide range of language abilities. Another advantage of the task is that it provides fine-grained information with a relatively short test. The SPELT-3 has been used in the past in studies testing the relationship between rhythm and grammatical skills (e.g., Gordon et al., 2015). Children’s verbal responses were scored for accuracy, according to procedures outlined in the test manual. Age-normed standard scores were used as a variable in the analysis.

#### Nonverbal intelligence

We measured nonverbal IQ using the Primary Test of Nonverbal Intelligence (PTONI; Ehrler & McGhee, 2008) to control for individual differences in nonverbal ability when testing associations between main study variables. In the PTONI children are presented with pictures and are asked to identify which picture does not belong with others. Age-normed standard scores were used in the analyses.

#### Questionnaires

One parent of each child was asked to complete a demographic questionnaire at the screening visit; this included the mother’s highest level of education (used as a proxy for socioeconomic status, or SES). This variable was scored on a scale ranging from 1 to 9 (see corresponding categories in Supplementary Material 1 in the Supporting Information). Parents also completed a questionnaire about children’s musical training. The questionnaire was adapted from previous studies (Chern et al., 2018; Gordon et al., 2015; Lense & Dykens, 2016), and it included five yes–no questions about musical training in school and out-of-school environments. The sum of yes answers, which could range from 0 to 5, was used as a measure of musical training for each participant in the analyses (see the musical training questionnaire in Supplementary Material 2).

#### Receptive grammar (exploratory analysis)

Previous research shows associations between receptive grammar and rhythm abilities (Lee et al., 2020; Swaminathan & Schellenberg, 2020). As we had data on receptive grammar measured by the TOLD-P:4 Syntactic Understanding subtest from all children with DLD and a subset of children with TD (*n* = 62) as a part of screening, we decided to run an exploratory analysis to test the association of receptive grammar with tapping measures. In this task children are presented with sentences testing various grammatical structures and three pictures with each sentence and directed to point to the picture that best represents the sentence. Scaled scores were used in the analysis.

### Data Analysis

Prior to data analysis, we examined the distribution of tapping data by calculating skewness and kurtosis for each tapping measure for the DLD and TD groups separately. We log-transformed those tapping measures that had a skewness lower than −2 or higher than 2 and/or a kurtosis lower than −4 or higher than 4 (see descriptive statistics in Table S3) to reach normal distribution. Data for both groups were log-transformed even if one of the groups passed our normality criteria. Based on these criteria, SMT, entrainment-region width, and slowest motor tempo measures were log-transformed, while fastest motor tempo measures were not log transformed. (We repeated all analyses after log-transforming fast tempo as well, and results showed the exact same pattern as with our original analysis.) Data showed a normal distribution after the transformations, therefore we decided not to exclude outliers. Nevertheless, we ran sensitivity analyses after excluding participants performing 2 standard deviations above or below the group mean in each group for the study’s main findings to ensure that our primary results were not driven by outliers. All results reported below remained significant and, in fact, in most cases became stronger after excluding outliers. (See results of the sensitivity analysis in Supplementary Material 3.)

We also examined whether the tapping measures showed equal variance across the TD and DLD groups to evaluate whether the data meet the assumption for equal variance required by analyses of variance (ANOVA) and analysis of covariance (ANCOVA). Standard deviations of tapping values were similar in the two groups for all tapping measures (Table S3) and equal variance in groups was confirmed with Bartlett tests (*p*_SMT_ = 0.078, *p*_ERW_ = 0.327, *p*_Fastest_ = 0.830, *p*_Slowest_ = 0.716).

To examine group differences between children with DLD and TD for the tapping measures (SMT, entrainment-region width, fastest motor tempo, slowest motor tempo), we conducted a one-way ANOVA for each tapping measure with group (TD vs. DLD) as a between subject factor. Significant group differences were further investigated with two ANCOVAs. Since children with TD had significantly higher nonverbal IQ (*F*(1, 120) = 18.50, *p* < 0.001, η^2^ = 0.13), we added it as a covariate in the first model. The two groups also showed a difference in musical training (*F*(1, 122) = 6.68, *p* = 0.011, η^2^ = 0.05); TD children completed significantly more musical training compared to children with DLD. Therefore, in a second model, both nonverbal IQ and musical training were added as covariates to test if the group difference is present after accounting for the effect of covariates.

We examined the relation of tapping measures with rhythm discrimination and expressive grammar performance as well as with receptive grammar performance in an exploratory analysis with Pearson correlations. Because we tested a reasonably small number of hypothesis-driven associations, we did not apply a multiple test correction to the confirmatory analyses. However, we did adjust exploratory analyses for multiple tests by applying a Bonferroni correction. The relations between these variables could be affected by individual differences in DLD status, age, nonverbal IQ, SES of the family, or musical training. Therefore, significant correlations were further investigated with two multiple linear regression models to test the effect of these covariates.

In the first set of models, each of the tapping measures that were significantly correlated with the dependent variable (rhythm discrimination for hypothesis 2, expressive grammar for hypothesis 3) were included in a regression with all general covariates (age, nonverbal IQ, SES, DLD status). In the second set of models musical training was included as an additional covariate. The first set of models will demonstrate whether the results stand when controlling for relevant cognitive and demographic measures and the second models will help us understand the role of musical training in these associations. Note that because of missing values, the number of data points in the analyses including covariates was slightly lower than in the analyses without covariates. Analyses were conducted using R (Version 3.6.0; R Core Team, 2017). The following packages were used to analyze and visualize data: psych (Revelle, 2019), Hmisc (Harrell & Dupont, 2019), car (Fox et al., 2019), effectsize (Ben-Shachar et al., 2020), ggplot2 (Wickham et al., 2016), and pROC (Robin et al., 2011).

## RESULTS

Descriptive statistics for all numeric study variables in the DLD and TD groups are displayed in Table S3 in the Supporting Information. Correlations between all study variables in the combined group of children with DLD and TD are displayed in Table S4.

### Slowest Motor Tempo Is Faster in Children With DLD vs. TD

**Figure 3.**
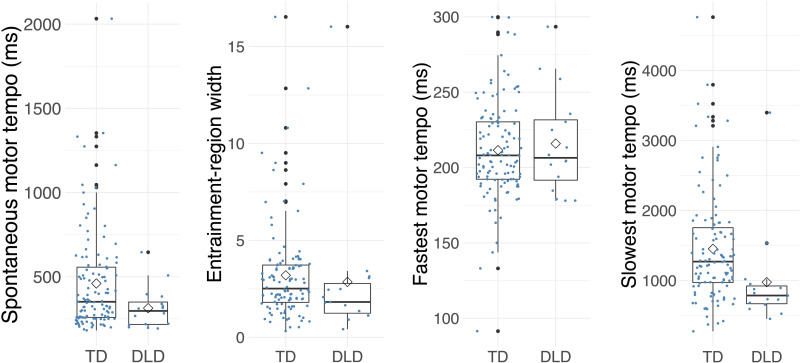
Spontaneous motor tempo (SMT), entrainment-region width, fastest motor tempo, and slowest motor tempo in typically developing (TD) children and children with developmental language disorder (DLD). Children with DLD show a faster SMT compared to TD children but the difference is not significant after controlling for covariates. There was no group difference in entrainment-region width or fastest motor tempo. Children with DLD show a faster slowest motor tempo compared to TD children even after controlling for nonverbal IQ.

To test our first hypothesis, we examined group differences between the TD and DLD groups in the tapping test conditions. Children with DLD showed a significantly faster SMT compared to TD children (*F*(1, 128) = 4.87, *p* = 0.029, η^2^ = 0.04), but there was no significant difference in entrainment-region width between the DLD and TD groups (*F*(1, 125) = 2.10, *p* = 0.15, η^2^ = 0.02). Follow-up analyses on slowest and fastest motor tempo (measures of the upper and lower limits of entrainment region) revealed that children with DLD also showed a faster slowest motor tempo than children with TD (*F*(1, 125) = 8.23, *p* = 0.005, η^2^ = 0.06), while fastest motor tempo did not show a group difference (*F*(1, 128) = 0.26, *p* = 0.614, η^2^ = 0.002; Figure 3).

To investigate whether group differences in SMT and slowest motor tempo would still be present even while controlling for the nonverbal IQ and musical training variables that also showed a group difference, we ran ANCOVAs (separately for each tapping measure). In the first set of analyses, we only added nonverbal IQ as a covariate (i.e., the only potential general covariate that showed a group difference). In the second set of models, we added musical training as an additional covariate. The group difference in SMT was not significant when nonverbal IQ (*F*(1, 119) = 2.02, *p* = 0.160, η^2^ = 0.04) or both covariates (*F*(1, 112) = 0.93, *p* = 0.337, η^2^ = 0.03) were added. In contrast, the group difference in slowest motor tempo was significant even when nonverbal IQ was added as a covariate (*F*(1, 116) = 4.25, *p* = 0.042, η^2^ = 0.07). The group difference in slowest motor tempo did not reach significance when both nonverbal IQ and musical training were added as covariates (*F*(1, 109) = 3.19, *p* = 0.077, η^2^ = 0.10).

### Children With Wider Entrainment Region Are More Accurate at Rhythm Discrimination

To test our second hypothesis, we investigated associations between tapping measures and rhythm discrimination performance. The width of the entrainment region was significantly correlated with rhythm discrimination (*r* = 0.33, *p* < 0.001), while SMT was not (*r* = −0.03, *p* = 0.73). Follow-up analyses on slowest and fastest motor tempo (measures of the upper and lower limits of entrainment region) showed that both fastest and slowest motor tempo were significantly correlated with rhythm discrimination performance (fastest motor tempo: *r* = −0.26, *p* = 0.004, slowest motor tempo: *r* = 0.28, *p* = 0.002). A faster fastest motor tempo (lower limit of entrainment region) and slower slowest motor tempo (upper limit of entrainment region) was associated with more accurate rhythm discrimination.

**Figure 4.**
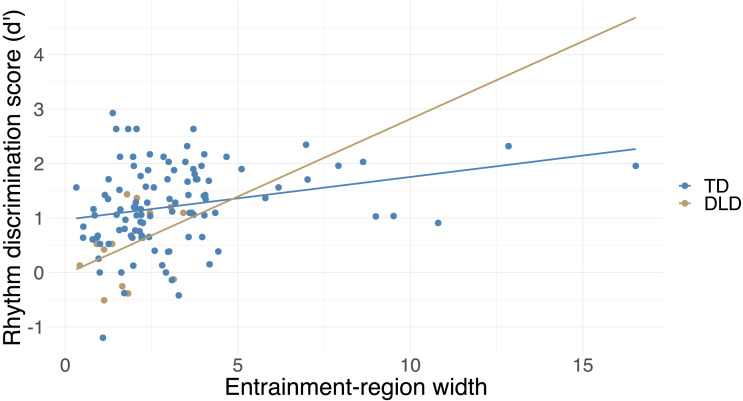
Rhythm discrimination scores are significantly associated with the width of the entrainment region after taking into account covariates (DLD status, age, SES, nonverbal IQ, musical training). The plot shows the relationship between rhythm and entrainment-region width with regression lines separately for the TD and DLD groups (i.e., without controlling for the effect of other covariates). DLD = developmental language disorder, SES = socioeconomic status, TD = typically developing.

The correlation between entrainment-region width and rhythm discrimination was significant after adding general covariates as well as after adding musical training as a covariate (Table 2 and Figure 4). Fastest and slowest motor tempo no longer showed an association with rhythm discrimination after adding general covariates, or both general covariates and musical training as covariates (Table 2).

**Table 2.** Multiple linear regression models testing associations between rhythm discrimination performance and entrainment-region width, fastest motor tempo, and slowest motor tempo.

Measure	Model 1		Model 2	
	β	*p*	β	*p*
**Entrainment-region width**				
Entrainment-region width	0.22	0.020	0.21	0.030
DLD status	−0.72	0.015	−0.71	0.021
Age	0.31	0.001	0.30	0.002
SES	0.04	0.638	0.04	0.681
Nonverbal IQ	−0.01	0.947	0.00	0.988
Musical training	–	–	0.03	0.734
Model fit	*F*(5, 105) = 6.38, *p* < 0.001, *R*^2^_adj_ = 0.197		*F*(6, 104) = 5.29, *p* < 0.001, *R*^2^_adj_ = 0.190	
**Fastest motor tempo**				
Fastest motor tempo	−0.08	0.443	−0.08	0.446
DLD status	−0.79	0.006	−0.72	0.015
Age	0.31	0.002	0.30	0.004
SES	0.07	0.398	0.06	0.494
Nonverbal IQ	0.01	0.880	0.03	0.776
Musical training	–	–	0.08	0.387
Model fit	*F*(5, 108) = 5.27, *p* < 0.001, *R*^2^_adj_ = 0.159		*F*(6, 107) = 5.62, *p* < 0.001, *R*^2^_adj_ = 0.157	
**Slowest motor tempo**				
Slowest motor tempo	0.15	0.114	0.14	0.171
DLD status	−0.74	0.014	−0.72	0.021
Age	0.33	<0.001	0.32	0.001
SES	0.03	0.730	0.03	0.773
Nonverbal IQ	−0.03	0.768	−0.02	0.846
Musical training	–	–	0.05	0.636
Model fit	*F*(5, 105) = 5.62, *p* < 0.001, *R*^2^_adj_ = 0.174		*F*(6, 104) = 4.69, *p* < 0.001, *R*^2^_adj_ = 0.167	

*Note*. Model 1 includes DLD status, Age, SES and nonverbal IQ as covariates, while Model 2 also includes musical training as a covariate. DLD = developmental language disorder, SES = socioeconomic status.

In the case of entrainment-region width, which showed a significant association with rhythm discrimination even after adding covariates, we tested whether the strength of the relation between entrainment-region width and rhythm discrimination differed between the TD and DLD groups. We used an ANCOVA in order to determine if the two regression lines have different slopes in the two groups. The ANCOVA showed the main effect of entrainment-region width (*F*(1, 115) = 14.65, *p* < 0.001) and DLD status (*F*(1, 115) = 7.02, *p* = 0.009) similarly to the original model, but no Entrainment-Region Width * DLD interaction (*F*(1, 115) = 0.11, *p* = 0.740), indicating no significant difference in the relation between entrainment-region width and rhythm discrimination between the two groups.

### No Associations With Expressive Grammar After Controlling for DLD Status

To test our third hypothesis, we investigated associations between the tapping measures and expressive grammar performance. Expressive grammar was significantly correlated with the width of the entrainment region (*r* = 0.20, *p* = 0.024) but not with SMT (*r* = 0.05, *p* = 0.553). Follow-up analyses on slowest and fastest motor tempo (measures of the upper and lower limit of entrainment region) showed a significant correlation between expressive grammar and slowest motor tempo (*r* = 0.23, *p* = 0.011), but fastest motor tempo was not associated with expressive grammar (*r* = 0.05, *p* = 0.582). Neither entrainment-region width nor slowest motor tempo were associated with expressive grammar performance when covariates were added to the model (Table 3).

**Table 3.** Multiple linear regression models testing associations of expressive grammar performance with entrainment-region width and slowest motor tempo.

Measure	Model 1		Model 2	
	β	*p*	β	*p*
**Entrainment-region width**				
Entrainment-region width	0.09	0.289	0.10	0.238
DLD status	−1.93	<0.001	−1.95	<0.001
Age	−0.01	0.868	−0.02	0.796
SES	0.00	0.990	0.00	0.963
Nonverbal IQ	0.09	0.270	0.09	0.307
Musical training	–	–	−0.03	0.761
Model fit	*F*(5, 108) = 16.64, *p* < 0.001, *R*^2^_adj_ = 0.409		*F*(6, 106) = 13.88, *p* < 0.001, *R*^2^_adj_ = 0.408	
**Slowest motor tempo**				
Slowest motor tempo	0.02	0.792	0.03	0.771
DLD status	−1.96	<0.001	−1.98	<0.001
Age	0.00	0.966	−0.01	0.871
SES	0.00	0.952	0.00	0.993
Nonverbal IQ	0.09	0.294	0.09	0.324
Musical training	–	–	−0.01	0.916
Model fit	*F*(5, 108) = 16.26, *p* < 0.001, *R*^2^_adj_ = 0.403		*F*(6, 106) = 13.50, *p* < 0.001, *R*^2^_adj_ = 0.401	

*Note*. Model 1 includes DLD status, Age, SES and nonverbal IQ as covariates, while Model 2 also includes musical training as a covariate. DLD = developmental language disorder, SES = socioeconomic status.

### Exploratory Analysis 1: Children With Wider Entrainment Region Have Enhanced Receptive Grammar Skills

Receptive grammar performance was significantly correlated with the width of the entrainment region (*r* = 0.36, *p* = 0.002) but not with SMT (*r* = 0.02, *p* = 0.843). Follow-up analyses on slowest and fastest motor tempo (measures of the upper and lower limits of entrainment region) showed a significant correlation with slowest motor tempo (*r* = 0.38, *p* < 0.001), but there was no correlation with fastest motor tempo (*r* = −0.08, *p* = 0.466). The correlation with entrainment-region width and slowest motor tempo was significant even at a Bonferroni corrected alpha level (0.05/4 = 0.013).

**Figure 5.**
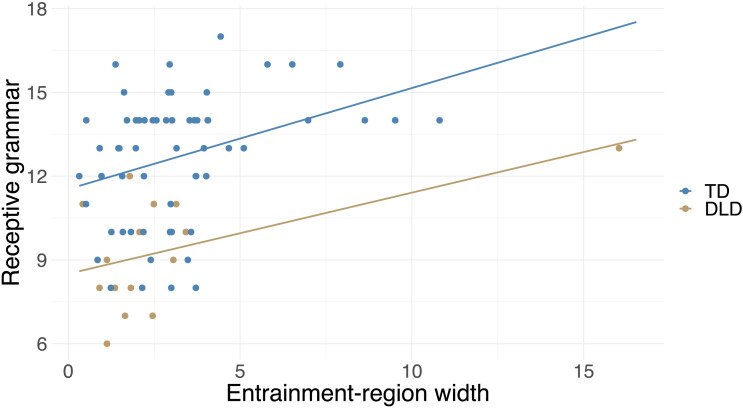
Receptive grammar scores are significantly associated with entrainment-region width after taking into account covariates. The plot shows the relationship between receptive grammar and entrainment-region width with regression lines separately for the typically developing (TD) and developmental languge disorder (DLD) groups (i.e., without controlling for the effect of other covariates).

We tested if these associations were still significant after controlling for general covariates and music training. The effect of entrainment-region width was significant after adding general covariates as well as after adding both general covariates and music training to the model (Table 4 and Figure 5). The effect of slowest motor tempo was significant after adding general covariates but not after adding musical training to the model (Table 4). Note that the visual inspection of Figure 5 suggests that the correlation was primarily driven by participants with a wide entrainment region and high performance on the receptive grammar test.

**Table 4.** Multiple linear regression models testing associations of receptive grammar performance with entrainment-region width and slowest motor tempo.

Measure	Model 1		Model 2	
	β	*p*	β	*p*
**Entrainment-region width**				
Entrainment-region width	0.33	0.003	0.30	0.010
DLD status	−1.34	<0.001	−1.27	<0.001
Age	−0.20	0.086	−0.22	0.063
SES	−0.05	0.665	−0.06	0.581
Nonverbal IQ	−0.07	0.549	−0.06	0.630
Musical training	–	–	0.11	0.378
Model fit	*F*(5, 57) = 8.65, *p* < 0.001, *R*^2^_adj_ = 0.381		*F*(6, 56) = 7.31, *p* < 0.001, *R*^2^_adj_ = 0.379	
**Slowest motor tempo**				
Slowest motor tempo	0.26	0.035	0.21	0.127
DLD status	−1.36	<0.001	−1.30	<0.001
Age	−0.18	0.131	−0.20	0.105
SES	−0.0	0.682	−0.05	0.639
Nonverbal IQ	−0.11	0.401	−0.09	0.520
Musical training	–	–	0.10	0.453
Model fit	*F*(5, 57) = 7.20, *p* < 0.001, *R*^2^_adj_ = 0.333		*F*(6, 56) = 6.05, *p* < 0.001, *R*^2^_adj_ = 0.328	

*Note*. Model 1 includes DLD status, Age, SES and nonverbal IQ as covariates, while Model 2 also includes musical training as a covariate. DLD = developmental language disorder, SES = socioeconomic status.

Since entrainment-region width still had a significant effect even after adding covariates, we tested with an ANCOVA whether there is a significant difference in the strength of the relation between entrainment-region width and receptive grammar between the TD and DLD groups by comparing the regression lines in the two groups. The main effect of entrainment-region width (*F*(1, 71) = 13.66, *p* < 0.001) and DLD (*F*(1, 71) = 23.48, *p* < 0.001) appeared similarly to the original model, but the interaction between entrainment-region width and DLD status was not significant (*F*(1, 71) = 0.01, *p* = 0.913), indicating no significant difference in the relation between entrainment-region width and receptive grammar between the two groups.

### Exploratory Analysis 2: Slowest Motor Tempo Can Differentiate Between Children With DLD and TD at an Acceptable Level

We performed a ROC (receiver operating characteristics) curve analysis to evaluate the performance of slowest motor tempo in differentiating between children with DLD and TD. The area under the ROC curve (AUC) is often used as a measure of classification accuracy. In this case, this value shows the probability that a randomly selected child from the TD group would show a slower (better) slowest motor tempo than a child randomly selected from the DLD group. We used 70% of the sample to train the model and 30% to test it. The AUC of slowest motor tempo was 0.75, which can be interpreted as an acceptable performance in predicting DLD status (values between 0.7 and 0.8 are considered acceptable, while values between 0.8 and 0.9 are considered excellent; Rice & Harris, 2005).

## DISCUSSION

The study investigated the relation of preferred tempo and entrainment-region width, measured with unpaced tapping tasks, with rhythm aptitude and expressive/receptive grammar in 5- to 7-year-old children with DLD and typical development. Children with DLD did not differ in SMT (measure of preferred tempo) or entrainment-region width compared to TD children, though children with DLD did not tap as slowly as TD children in the slowest motor tempo condition even after controlling for nonverbal IQ (i.e., children with DLD had a faster upper (slow) limit to their entrainment region compared to TD children). Rhythm discrimination was positively associated with entrainment-region width even after accounting for the effect of demographic and cognitive variables. In general, our results indicate that SMT, the measure previous studies primarily focused on, is less relevant in the context of DLD and rhythm development in young school-age children, whereas entrainment-region width, or at least its slower (upper) limit, taps into processes that differ between children with DLD and TD and children with more accurate and impaired rhythm aptitude. Although expressive grammar was not associated with tapping measures after controlling for covariates, receptive grammar showed a significant association with entrainment-region width and slowest motor tempo, revealed by an exploratory analysis.

Our first aim was to investigate whether children with DLD show a delayed development in preferred tempo and entrainment-region width. Children with DLD showed a faster SMT compared to TD children, although the difference was not significant after including nonverbal IQ as a covariate. Normalized entrainment-region width did not differ between the two groups, but slowest motor tempo (corresponding to the upper limit of the entrainment region) was faster for children with DLD than for TD children even after taking into account individual differences in nonverbal IQ. Drake et al. (2000) suggest that engagement with slower oscillators makes children able to process higher hierarchical levels in auditory stimuli such as music or spoken language (which they refer to as *future-oriented attending*). Following this idea, we speculate that a limited slowest motor tempo in children with DLD could be associated with their characteristic difficulties with syntactic processing as well as with rhythm processing difficulties. Note that slowest motor tempo could be affected by the strategy the child uses in this condition, which could lead to large differences independently of the child’s rhythm abilities. For instance, a child may choose to tap at an extremely slow pace without relying on internal time-keeping abilities. Although we cannot exclude this possibility, we do not have a reason to believe that strategies to complete the task significantly differed between children with TD and DLD, and therefore, our interpretation of this finding is that the group difference originated from differences in rhythm abilities.

In contrast with previous studies finding slower fastest tempo in children with DLD compared to TD children (Bishop, 2002; Leonard et al., 2007), in the current study, fastest motor tempo did not differ between DLD and TD groups. In Bishop (2002) children had to tap continually as fast as they could with a tally counter device with their thumb for 30 s and in Leonard et al. (2007) children had to tap continually on a key for 5 s. Pressing a key or moving a tally counter very quickly might be a more challenging motor task than tapping on an iPad. These methodological differences could account for the inconsistent results. According to Drake et al. (2000) fast tempo can be used as a proxy of fast oscillators that play a role in lower-level processing (which they refer to as *analytic attending*) that could be associated with processing of lower-level segments of language or music (tones, phonemes/syllables). It is, therefore, possible that processing of lower-level cues, such as the acoustic characteristics of phonemes and syllables, was intact in this sample of children with DLD in accordance with their fastest motor tempo not differing from that of the TD group. Since we did not have enough information on children’s phonological processing abilities, we could not directly test this hypothesis, and future research should test fastest motor tempo and different aspects of phonological processing in children with DLD to learn more about this potential relationship.

With respect to the upper (slow) limit of the entrainment region, note that the group (DLD vs. TD) difference became marginally significant when we controlled for the effect of both nonverbal IQ and musical training (although it is significant when outliers are excluded from the analysis; see Supplementary Material 3 in the Supporting Information). We have added nonverbal IQ and musical training in separate steps to the analysis because while nonverbal IQ is a potential confound when testing group differences (a group difference might appear because of the lower nonverbal IQ of the DLD group in the current study), it is unlikely in the case of music training. Music training could be a mediator, meaning that children with DLD have a reduced upper (slow) limit of entrainment region as a result of less music training (see Drake et al., 2000), although it is unclear if the very limited musical training in our sample of 5- to 7-year-olds can really account for such differences. It is also possible that there is no causal relationship between musical training, DLD, and slowest motor tempo, and that associations could be accounted for primarily by shared genetic architecture underlying language skill, musical training, and entrainment-region width. For instance, results of a recent twin study (Wesseldijk et al., 2021) suggest that associations between music training and verbal skills can be explained by individual differences in music aptitude, that are partly driven by familial effects, suggesting shared genetic predispositions between music training and verbal skills. Another recent family-based study (Gustavson et al., 2021) also suggests a genetic link between music engagement (skills, interest, lessons) and language development, in line with the framework proposed by Nayak et al. (2022). The interplay between genetic and environmental factors in the relationships between DLD status, musical training, and slowest motor tempo should be investigated in the future.

Studies with children with DLD generally show slower reaction times in cognitive tasks compared to TD children (Kail, 1994). This was proposed to be the result of slower processing speed. Importantly, our results on faster slowest motor tempo and equally fast SMT in children with DLD as in TD children do not contradict this general pattern in the literature. Although cognitive speed may influence inter-tap intervals in these conditions, performance would be influenced primarily by the characteristics of internal oscillators according to the DAT. In addition, no cognitive computations need to be completed between each tap; therefore, reduced processing speed would not lead to slower tapping in an unpaced tapping task. The lack of significant group difference in fast tempo, however, contradicts some of the previous findings, and potential methodological explanations for these differences are discussed above.

Reduced upper (slow) limit of the entrainment region, measured by slowest motor tempo, in children with DLD is in line with the Atypical Rhythm Risk Hypothesis that posits that individuals with atypical rhythm abilities are at a higher risk for speech-language disorders (Ladányi et al., 2020). Ladányi et al. (2020) argued that if the Atypical Rhythm Risk Hypothesis is supported by sufficient evidence, rhythm abilities could be used in the screening and treatment of children with speech-language disorders. The assessment of the upper limit of the entrainment region using slow tapping offers a potential avenue for early identification and novel treatment approaches in children with DLD. Children with DLD are often under-identified and underserved (McGregor, 2020), even though childhood communication disorders have broad-ranging impacts on academic, social, and vocational outcomes later in life (Rosenbaum & Simon, 2016). Tapping tasks are quick and inexpensive and could be administered to preschoolers and school-aged children by various professionals (teachers, nurses, school counselors, pediatricians) who do not have specialized speech–language pathology expertise. Thus, these tasks have the potential for wide dissemination to screen children to obtain a clinically relevant indicator of risk for DLD, attributable to co-morbid impairments in rhythmic processing and language development (see Ladányi et al., 2020). To explore this possibility based on the current data, we evaluated how well children can be classified into TD and DLD categories based on their slowest motor tempo performance. The AUC was 0.75, which can be interpreted as acceptable classification performance of the model. We also checked the ratio of children in the two groups who show 1 standard deviation faster slowest motor tempo than the mean in TD children, and we visually examined individual performances (Figure 3). In the DLD group 47% of the children fell under the cutoff point, and most of the other children in the group were quite close to it, while in the TD group 15% of the children fell under the cutoff. These results are promising as a first step toward proof of concept; importantly, future clinical research in larger samples should test if assessing unpaced tapping—potentially in combination with other rhythm tasks—yields an appropriate specificity and sensitivity as a screener for DLD. In addition to screening, such tasks may also serve to identify children with shared rhythmic and language deficits who may benefit from treatment approaches that include a rhythmic element (Schön & Tillmann, 2015).

Reduced upper (slow) limit of entrainment region in children with DLD could be also be relevant for recommendations on speech rate when talking to children with DLD. From a speech processing perspective, it is reasonable to consider that one might talk slowly to children with DLD to make language processing easier for them, and there is some research evidence as well for the negative effect of fast speech rate on speech processing of children with DLD (Guiraud et al., 2018). However, based on the faster slowest motor tempo of children with DLD in the present study suggests that children with DLD have particular problems with processing regularities in stimuli that appear at a slow pace. Thus, speaking very slowly to them might hinder their syntactic processing. For instance, inserting pauses between sentences could be more beneficial than pronouncing words slowly, as it could help to compensate slow processing speed without making the duration of sentences longer. Importantly, future research should examine the effect of slow and fast speaking rate on language comprehension of children with DLD before such recommendations are proposed. It is also possible that different children would benefit from different strategies, and that performance on a slowest motor tempo task could be a factor in personalizing speech-language therapy.

More generally, we hypothesized that SMT and entrainment-region width would be associated with rhythm aptitude. Supporting this hypothesis, rhythm discrimination performance was associated with entrainment-region width, even after accounting for the effect of age, DLD status, SES, nonverbal IQ, and music training. None of the other tapping measures was associated with rhythm discrimination after adding age, DLD status, SES, and nonverbal IQ to the model. Following the framework of Drake et al. (2000), this result suggests that rhythm aptitude is associated with focal attending, that is, the ability to focus attention away to multiple hierarchical levels higher and lower than the preferred tempo.

Contrary to our prediction, none of the tapping measures were associated with expressive grammar after adding age, DLD status, SES, and nonverbal IQ to the model, with DLD status having a particularly strong effect. If slowest motor tempo mirroring engagement with slow oscillators supports processing of structures at high levels of the syntactic hierarchy, then one would expect that expressive grammar is associated with slowest motor tempo and entrainment-region width. The lack of these associations does not support the role of the upper (slow) limit of entrainment region in expressive syntactic development.

A limitation of the current study is the sample size of the DLD group which could account for the lack of associations between expressive grammar and tapping measures. DLD is a spectrum disorder with multiple weaknesses, and the strengths of various impairments show a high heterogeneity in the population. It is possible that each impairment is weakly related to tapping measures (slowest motor tempo or entrainment-region width), and thus related to weaker language skills, but the effect will not be present if associations with only one construct is measured. Future work should test various constructs often impaired in DLD (both receptive and expressive grammar and vocabulary, phonological awareness, and comorbidities such as motor and attentional problems) in larger samples. By applying modeling approaches this work could shed light on associations between the limits of entrainment region and impairments in DLD. A preliminary supporting result for this idea was demonstrated in an exploratory analysis in the current study showing a positive association between entrainment-region width and receptive grammar, measured by performance on the Syntactic Understanding subtest of the TOLD-P:4. This association was still present even after including age, DLD status, SES, nonverbal IQ, and musical training as covariates.

Although the effect of musical training was not one of the main research questions in the current study and the study sample did not allow for a thorough investigation of the question due to the limited amount of musical training in the 5- to 7-year-old age range, some interesting associations can be observed in the results. The correlations we report in Table S4 and the regression models show that children with more musical training have a wider entrainment region with a higher upper (slow) limit, higher rhythm aptitude, and better receptive grammar skills. The causal relationship between these factors is yet unknown with some evidence both for (Flaugnacco et al., 2015) and against (Sala & Gobet, 2020; Wesseldijk et al., 2021) a causal relationship between musical training and language abilities.

The two earlier studies that tested preferred tempo, entrainment-region width, and slowest and fastest motor tempo proposed intriguing conceptual models in the framework of the DAT (Drake et al., 2000; McAuley et al., 2006). They did not aim to give, however, a neural account for the models. Recent work emphasizes the role of neural entrainment in the processing of musical rhythm and spoken language stimuli. This body of work strongly suggests that neural oscillations entrain with regularities in musical rhythm and spoken linguistic stimuli at multiple hierarchical levels (tones/syllables, beats/words, metrical units/phrases) and has shown that more efficient entrainment is associated with more accurate behavioral performance (Ding et al., 2015; Fiveash et al., 2021; Giraud et al., 2007; Giraud & Poeppel, 2012; Henke & Meyer, 2021; Large et al., 2015; Meyer, 2018; Peelle & Davis, 2012; Poeppel & Assaneo, 2020; Tal et al., 2017). For instance, Henke and Meyer (2021) propose that processing cycles in the delta frequency band constrain the length of syntactic phrases we can process. Based on this work, it is plausible that the characteristics of neural oscillations serve as the neural basis of preferred tempo and entrainment-region width.

The idea that neural oscillations could be related to preferred tempo and entrainment-region width is supported by emerging neurobiological evidence. One study (Bauer et al., 2015) tested associations between preferred tempo and various types of resting state and task-related oscillatory activities and found an association with the frequency of beta oscillations appearing for motor activity. More specifically, participants’ preferred tempo was roughly eight times slower than the frequency of their motor-related beta oscillations. Furthermore, Michaelis et al. (2014) demonstrated that the excitability of the motor cortex was highest when listening to stimuli with a beat rate at the individual’s preferred tempo (confirmed both by SMT and preferred perceptual tempo) compared to faster and slower tempi. These neurobiological findings are consistent with the idea of endogenous oscillators underlying preferred tempo and, by extension, entrainment-region width.

Several studies have shown that the motor system influences the perception of sound, even when no overt movement occurs, and it has been proposed that the motor cortex may act as a neural oscillator guiding auditory processing (for a review see Poeppel & Assaneo, 2020). The role of beta oscillations has been emphasized in rhythm processing potentially due to their role in long-range intracortical interactions, while delta oscillations generated by the motor cortex were proposed to play a role in optimizing the parsing, encoding, and processing of slow linguistic information (Arnal & Giraud, 2012; Morillon et al., 2019). These results are consistent with the idea that oscillatory activity in the beta and delta band is related to preferred tempo and entrainment-region width measured by unpaced tapping tasks. Although the exact mechanisms underlying unpaced comfortable, fast and slow tapping are still to be understood, these results suggest that intrinsic oscillatory activity underlies the tempo participants tap within this task.

Regarding future neurobiological research, the current results point toward the importance of assessing an individual’s entrainment-region width, in particular its upper (slowest tempo) limit. Future studies could test individual differences in the characteristics of low delta oscillatory activity and its relationship with spoken language and rhythm processing as well as impaired slow neural oscillatory mechanisms (e.g., neural entrainment) in the low delta band as one of the neurobiological correlates of atypical grammar and rhythm development in children with DLD. DLD is a multifactorial disorder with various strengths and weaknesses that can also differ across children with DLD, and we propose that an impairment in the upper limit of the entrainment region could be one factor associated with DLD in at least some children with DLD (see also Lense et al., 2021). Although to our knowledge, neural entrainment to rhythmic or spoken language stimuli has not been tested yet in children with DLD, results from children with dyslexia (often comorbid with DLD) show less accurate neural entrainment to rhythmic stimuli compared to TD children (Colling et al., 2017).

## SUMMARY AND CONCLUSIONS

The current study investigated preferred tempo and entrainment-region width measured with unpaced tapping at different tempi in children with DLD and TD and their associations with rhythm aptitude and expressive grammar abilities. Reduced upper limit of entrainment region in children with DLD vs. TD (corresponding to a faster slowest motor tempo) suggests that children with DLD are at an earlier phase in the development of motor tempi potentially due to the delayed/atypical development of oscillatory mechanisms that also underlie rhythm and language development. The association between entrainment-region width and rhythm aptitude suggests shared mechanisms underlying unpaced tapping and rhythm processing, which is consistent with the idea that intrinsic neural oscillations play a role in both processes. These results have a theoretical relevance and motivate further research on entrainment-region width and its upper (slow) limit, in addition to preferred tempo, which most of the studies have focused on so far. In addition, the unique variance that the upper limit of the entrainment region (measured with slowest motor tempo) explained in DLD status raises the possibility that assessing slowest motor tempo could be integrated in screenings for DLD, a possibility that could be studied in future work.

## ACKNOWLEDGMENTS

We thank the families for participating, and we thank the following individuals for assistance with data collection and data management: Ashley Hirsch, Karli Oxford-Jordan, Alaina Baird, Mari McCarville, Rebecca Embalabala, Alexander Chern, Courtney Walters, Leah Boyd, Kristin Gummersheimer, Cara Petrucci, Aysu Erdemir, Catherine Bush, Youjia Wang. We thank Navya Thakkar for her assistance with figures. We thank the three anonymous reviewers for their insightful comments and suggestions.

This project was supported by funding from the National Institute on Deafness and Other Communication Disorders of the National Institutes of Health, the Vanderbilt Trans-Institutional Program grant, the Vanderbilt Institute for Clinical and Translational Research (VICTR), and the Vanderbilt Clinical Translational Science Award (CTSA) grant from the National Center for Advancing Translational Sciences (NCATS)/NIH. The content is solely the responsibility of the authors and does not necessarily represent the official views of the National Institutes of Health.

## FUNDING INFORMATION

Reyna L. Gordon, National Institute on Deafness and Other Communication Disorders of the National Institutes of Health (https://dx.doi.org/10.13039/100000055), Award ID: R01DC016977. Reyna L. Gordon, National Institute on Deafness and Other Communication Disorders of the National Institutes of Health (https://dx.doi.org/10.13039/100000055), Award ID: K18DC017383. Reyna L. Gordon, National Institute on Deafness and Other Communication Disorders of the National Institutes of Health (https://dx.doi.org/10.13039/100000055), Award ID: R03DC014802. Reyna L. Gordon, Vanderbilt Trans-Institutional Program grant. Reyna L. Gordon, Vanderbilt Institute for Clinical and Translational Research (VICTR) grant. Gordon Bernard, Vanderbilt CTSA grant from the National Center for Advancing Translational Sciences (NCATS)/NIH, Award ID: ULTR002243.

## AUTHOR CONTRIBUTIONS

**Enikő Ladányi**: Conceptualization: Lead; Data curation: Lead; Formal analysis: Lead; Investigation: Lead; Methodology: Equal; Visualization: Lead; Writing—original draft: Lead; Writing—review & editing: Lead. **Michaela Novakovic**: Methodology: Supporting; Software: Supporting; Writing—original draft: Supporting. **Olivia A. Boorom**: Data curation: Equal; Investigation: Lead; Methodology: Equal; Project administration: Lead; Writing—original draft: Supporting; Writing—review & editing: Supporting. **Allison S. Aaron**: Investigation: Supporting; Methodology: Supporting; Project administration: Supporting. **Alyssa C. Scartozzi**: Data curation: Supporting; Formal analysis: Supporting; Methodology: Supporting; Project administration: Supporting; Writing—original draft: Supporting. **Daniel E. Gustavson**: Formal analysis: Equal; Methodology: Equal; Writing—original draft: Supporting. **Rachana Nitin**: Data curation: Supporting; Investigation: Supporting; Visualization: Supporting; Writing—original draft: Supporting. **Peter O. Bamikole**: Investigation: Supporting; Methodology: Supporting. **Chloe Vaughan**: Investigation: Supporting; Methodology: Supporting; Project administration: Supporting. **Elisa Kim Fromboluti**: Investigation: Supporting; Methodology: Supporting. **C. Melanie Schuele**: Conceptualization: Supporting; Funding acquisition: Supporting; Methodology: Supporting; Writing—original draft: Supporting; Writing—review & editing: Supporting. **Stephen M. Camarata**: Conceptualization: Supporting; Funding acquisition: Supporting; Methodology: Supporting; Writing—original draft: Supporting. **J. Devin McAuley**: Conceptualization: Equal; Formal analysis: Supporting; Funding acquisition: Supporting; Methodology: Equal; Supervision: Equal; Writing—original draft: Supporting; Writing—review & editing: Supporting. **Reyna L. Gordon**: Conceptualization: Equal; Formal analysis: Supporting; Funding acquisition: Lead; Investigation: Supporting; Methodology: Equal; Project administration: Lead; Software: Supporting; Visualization: Supporting; Writing—original draft: Supporting; Writing—review & editing: Supporting.

## DATA AVAILABILITY STATEMENT

The data and the analysis scripts that we used in the current study are available for research purposes at https://osf.io/cpeky/.

## TECHNICAL TERMS

Developmental language disorder (DLD): A developmental disorder that primarily affects language development without an obvious biomedical origin.
Rhythm abilities: Set of abilities associated with rhythm perception, including the perception of grouping, beat and meter.
Beat: A series of approximately periodic time points in music that stand out in some way to the listeners (i.e., they are accented).
Preferred tempo: Individual preference for a particular pace (tempo) of a sequence of elements.
Meter: The temporal organization of beats on hierarchically nested time scales.
Entrainment region: The range of tempi that affords attentional synchronization.
Spontaneous motor tempo (SMT): An individual’s preferred rate of rhythmic motor activity that has been proposed to measure preferred tempo.
