# Supplementary material for: Using Motor Tempi to Understand Rhythm and Grammatical Skills in Developmental Language Disorder and Typical Language Development
Source: Neurobiol Lang (Camb). Author manuscript; Available in PMC 2023 Mar 2. (PMC9979588; doi:10.1162/nol_a_00082)
Supplement: Supplementary file 1 [file NIHMS1873134-supplement-Supplement.pdf]

Supplementary information

Supplementary Table 1. Language characteristics of individual participants in the DLD group of the study

Participant	TOLD Semantic Index	TOLD Organizing Index	TOLD Speaking Index	TOLD Grammar Index	TOLD Listening Index	TOLD Spoken Language Index	TEGI Criterion Score
DLD1	108	92*	103	94*	108	101	Below*
DLD2	92*	86*	70*	70*	88*	78*	Below*
DLD3	82*	83*	79*	86*	91*	82*	Below*
DLD4	94*	80*	100	94*	102	93*	Above
DLD5	71*	72*	76*	78*	80*	72*	Below*
DLD6	112	94*	109	106	122	109	Below*
DLD7	71*	75*	79*	84*	83*	75*	Below*
DLD8	82*	80*	76*	90*	102	84*	Below*
DLD9	82*	80*	73*	80*	91*	78*	Below*
DLD10	80*	83*	97	106	100	92*	Below*
DLD11	82*	97	88*	108	100	94*	Above
DLD12	94*	86*	85*	86*	100	88*	Above
DLD13	77*	75*	79*	78*	83*	75*	Below*
DLD14	96	86*	103	109	119	103	Above
DLD15	96	83*	94*	90*	102	92*	Below*
DLD16	102	100	88*	92*	102	96	Below*

Note: Measures that qualified each child for the DLD group are marked with an asterisk.

Supplementary Table 2. TOLD-P:4 Picture Vocabulary, Sentence Imitation, and Morphological Completion subtest scores of typically developing children without a valid non-verbal IQ score

Participant	Picture Vocabulary	Sentence Imitation	Morphological Completion
TD1	11	12	11
TD2	12	11	13
TD3	11	9	11
TD4	13	9	11
TD5	15	15	15
TD6	15	14	10
TD7	15	15	10
TD8	12	12	13

Note: Scaled scores. Range: 1-20; 8-12 corresponds to average performance. Although these children with TD did not have a valid non-verbal IQ score, their average or above average language performance suggests that their non-verbal IQ was in the typical range.

Supplementary Table 3. Descriptive statistics for all study variables in the DLD and TD groups

Measure	N	M	SD	Min	Max	Skew	Kurtosis
**TD**							
SMT	114	460ms	299ms	181ms	2033ms	2.21	6.51
**SMT_{log}**	114	5.98	0.52	5.2	7.62	0.76	-0.09
Entrainment-region width	112	3.2	2.47	0.32	16.53	2.58	8.77
**Entrainment-region $width_{log}$**	112	0.94	0.66	-1.14	2.81	-0.09	0.73
**Fastest motor tempo**	114	212ms	32ms	91ms	300ms	0.06	1.46
Slowest motor tempo	112	1454ms	788ms	275ms	4763ms	1.44	2.49
**Slowest motor $tempo_{log}$**	112	7.15	0.52	5.62	8.47	-0.1	0.21
**Rhythm discrimination d'**	108	1.21	0.75	-1.2	2.93	-0.2	0.04
**SPELT standard score**	112	113.17	6.43	90	126	-0.63	0.77
**TOLD Sentence Understanding standard score**	62	12.73	2.3	8	17	-0.51	-0.65
**Age**	114	6.57	0.85	5.07	8.17	0.1	-1.28
**SES**	111	7.48	0.99	5	9	-0.05	-0.61
**Nonverbal IQ**	106	121.73	18.94	82	150	-0.4	-0.99
**Musical training**	110	1.16	1.05	0	4	0.73	-0.11
**DLD**							
SMT	16	313ms	124ms	193ms	645ms	1.21	0.78
**SMT_{log}**	16	5.68	0.35	5.26	6.47	0.58	-0.67
Entrainment-region width	15	2.86	3.75	0.42	16.03	2.85	7.21
**Entrainment-region $width_{log}$**	15	0.67	0.8	-0.87	2.77	0.67	1.17
**Fastest motor tempo**	16	216ms	34ms	178ms	293ms	0.76	-0.48
Slowest motor tempo	15	977ms	717ms	453ms	3399ms	2.53	5.76
**Slowest motor $tempo_{log}$**	15	6.74	0.48	6.12	8.13	1.46	2.04
**Rhythm discrimination d'**	14	0.54	0.68	-0.51	1.44	-0.18	-1.62
**SPELT standard score**	14	91.21	17.51	65	114	-0.24	-1.65
**TOLD Sentence Understanding standard score**	16	9.56	2.16	6	13	0.09	-1.3
**Age**	16	6.68	0.87	5.64	8.08	0.24	-1.7
**SES**	14	7	1.04	5	9	0	-0.66
**Nonverbal IQ**	16	100.81	10.88	83	122	0.07	-1.13
**Musical training**	14	0.43	0.51	0	1	0.26	-2.07

Note: For SMT, entrainment-region width and slowest motor tempo both non-log transformed and log-transformed values are included in the table. Measures used in the analyses are bolded.

Supplementary Table 4. Correlations Among All Study Measures

	1	2	3	4	5	6	7	8	9	10	11
1. SMT	1										
2. Entrainment-region width	-.35***	1									
3. Fastest motor tempo	.09	-.25**	1								
4. Slowest motor tempo	.41***	.7***	-.14	1							
5. DLD status	-.19*	-.13	.04	-.25**	1						
6. Rhythm discrimination	-.03	.33***	-.26**	.28**	-.28**	1					
7. Expressive grammar	.05	.20*	.05	.23*	-.64***	.33***	1				
8. Receptive grammar	.02	.36**	-.08	.38***	-.49***	.23	.5***	1			
9. Age	-.03	.2*	-.5***	.16	.04	.38***	-.06	-.03	1		
10. SES	.21*	.09	.17	.24**	-.15	.07	.1	.1	-.12	1	
11. Nonverbal IQ	.22*	.04	.08	.23*	-.37***	.06	.31***	.26*	-.14	.14	1
12. Musical training	.08	.29**	-.14	.34***	-.23*	.22*	.13	.28*	.25**	.15	-.07

Note: SMT, entrainment-region width and slowest motor tempo values are log-transformed.

* $p < .05$, ** $p < .01$, *** $p < .001$

Supplementary Material 1. Corresponding categories of the parent highest level of education scale

1 - Elementary: 0 years
2 - Elementary: 1 to 6 years
3 - Elementary: 7 to 9 years
4 - Elementary: 10 to 11 years
5 - Elementary: 12 years or GED
6 - College/Technical: 1-2 years
7 - College/Technical: 3-4 years
8 - Graduate or Professional School: 1-2 years
9 - Graduate or Professional School: 3-4+ years

Supplementary Material 2. Child musical training questionnaire

1. Individual instruction (not in school)?
Yes No

2. Group instruction (not in school)?
Yes No

3. Individual lessons in school?
Yes No

4. Group instruction in school?
Yes No

5. Has your child participated in a choir, band, orchestra, or other musical ensemble, either through their school or as an extra-curricular activity?
Yes No

Supplementary Material 3. Sensitivity analyses

In order to ensure that our main findings are not driven by outliers, we repeated these analyses with excluding participants performed 2SD below or above the group mean in each group.

Slowest motor tempo is faster in children with DLD vs. TD

After excluding outliers in slowest motor tempo (DLD: n = 1 due to extremely slow tempo, TD: n = 4 due to extremely fast tempo and n = 2 due to extremely slow tempo), we found a significantly faster slowest motor tempo in children with DLD compared to children with TD ($F(1,118) = 19.03$, $p <.001$, η2 = .14), which was present even when nonverbal IQ ($F(1,110) = 11.136$, $p = .001$, $\eta^2 = .14$) and both non-verbal IQ and musical training are added as a covariate (($F(1,104) = 6.073$, $p = .015$, $\eta^2 = .14$). Note that the effect sizes became larger than in the original analysis and slowest motor tempo has a significant effect even when musical training is included in the model while this effect became marginally significant in the original analysis.

Children with wider entrainment region are better at rhythm discrimination

After excluding outliers in entrainment-region width (DLD: n = 1 due to extremely wide entrainment region, TD: n = 3 due to extremely narrow entrainment region, n = 3 due to extremely wide entrainment region), we found a significant positive association between wider entrainment region and rhythm discrimination performance ($r = .33$, $p < .001$). Entrainment-region width had a significant effect even when general covariates ($p = .012$) or both general covariates and musical training ($p = .017$) were added to the model. Effect sizes were the same or slightly increased and p values became lower compared to the original analysis.

Children with wider entrainment region are better at receptive grammar

After excluding outliers in entrainment-region width (see above), we found a significant positive association between wider entrainment region and receptive grammar performance ($r = .43$, $p < .001$). Entrainment-region width had a significant effect even when general covariates ($p = .005$) or both general covariates and musical training ($p = .012$) were added to the model. Effect sizes were the same or slightly decreased and p values became slightly higher compared to the original analysis.
